# Spectroscopic ellipsometry for low-dimensional materials and heterostructures

**DOI:** 10.1515/nanoph-2022-0039

**Published:** 2022-04-18

**Authors:** SeokJae Yoo, Q-Han Park

**Affiliations:** Department of Physics, Inha University, Incheon 22212, Korea; Department of Physics, Korea University, Seoul 02841, Korea

**Keywords:** electronic structures, ellipsometry, low dimensional materials, permittivity, spectroscopy, van der Waals materials

## Abstract

Discovery of low-dimensional materials has been of great interest in physics and material science. Optical permittivity is an optical fingerprint of material electronic structures, and thus it is an important parameter in the study of the properties of materials. Spectroscopic ellipsometry provides a fast, robust, and noninvasive method for obtaining the optical permittivity spectra of newly discovered materials. Atomically thin low-dimensional materials have an extremely short vertical optical path length inside them, making the spectroscopic ellipsometry of low-dimensional materials unique, compared to traditional ellipsometry. Here, we introduce the fundamentals of spectroscopic ellipsometry for two-dimensional (2D) materials and review recent progress. We also discuss technical challenges and future directions in spectroscopic ellipsometry for low-dimensional materials.

## 1 Introduction

Reducing the dimensionality of materials enhances hidden quantum effects and introduces unique properties that are absent in higher-dimensional materials. Many interesting physics, such as superconductivity [1, 2], valley polarization [3, 4], and charge density waves [5], have been explored using low-dimensional materials. These achievements have led to the intense exploration of new low-dimensional materials. Theoretical computations have predicted more than 4000 two-dimensional (2D) materials [6], but most of them await experimental investigation. In addition, strong interlayer interactions in low- and mixed-dimensional heterostructures dominate the entire electronic structure, leading to unconventional optical, electronic, and magnetic properties that were absent in each constituent. Numerous 2D material candidates and their heterostructure combinations present countless possibilities, requiring a fast, robust, and noninvasive method for characterizing the electronic structures of newly discovered low-dimensional materials.

**Figure 1:**
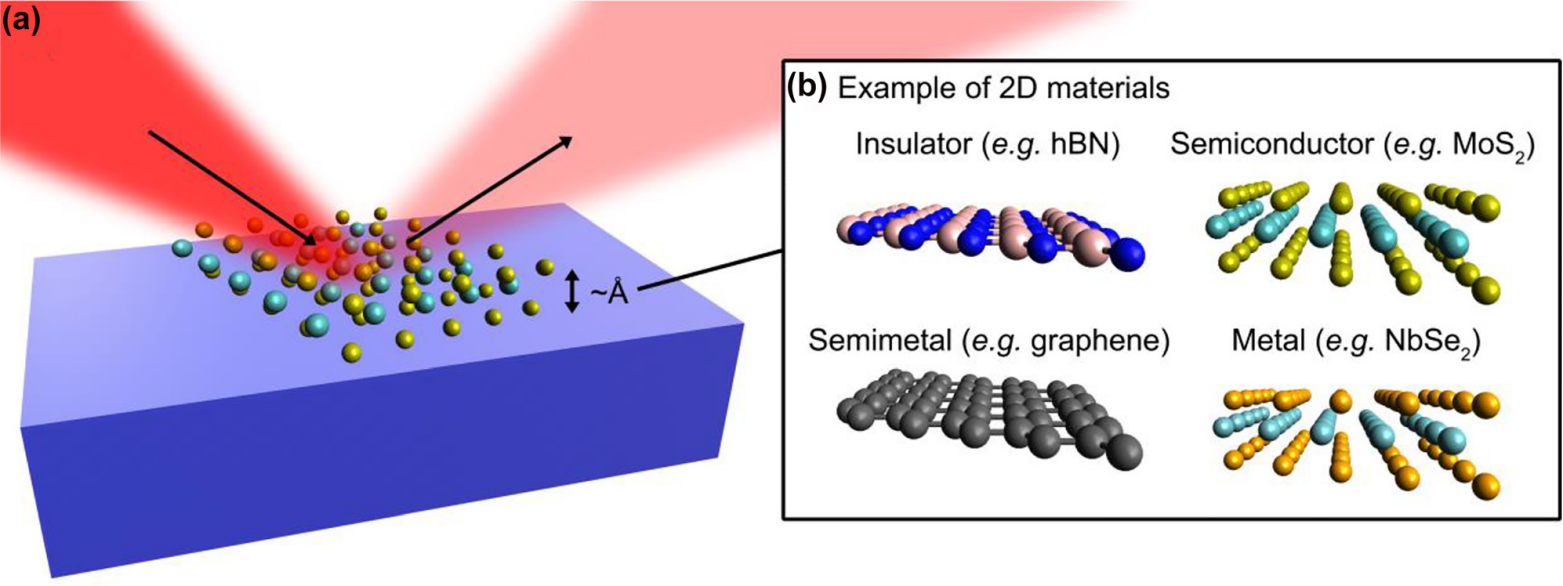
Spectroscopic ellipsomety for low-dimensional materials. (a) Spectroscopic ellipsometry measures amplitude and phase of the light obliquely reflected by 2D materials on a substrate. Thickness of the 2D materials is usually at the Angstrom scale. (b) Typical 2D materials with various optical, electrical, and magnetic properties.

Spectroscopic ellipsometry, an optical technique that measures complex optical permittivity spectra using elliptically polarized and obliquely incident light (Figure 1a), is a popular method for obtaining information about the electronic structures of materials using permittivity. Spectroscopic ellipsometry can be implemented straightforwardly in a laboratory and is also available using a commercial equipment. Further, it is a noninvasive technique as the light intensity does not cause thermal damage to the sample at the appropriate intensity. Many efforts have been made to obtain the permittivity of 2D materials using conventional spectroscopic ellipsometry. However, only a few studies have been conducted to develop novel spectroscopic ellipsometry techniques specifically designed for atomically thin 2D materials because the small optical path length inside them makes ellipsometry unique compared to conventional ellipsometry. Low-dimensional spectroscopic ellipsometry can provide a deterministic permittivity measurement that does not require parameter fitting, whereas conventional ellipsometry generally requires parameter fitting and an arbitrary choice of spectral lineshape functions.

In this article, we review the fundamentals of spectroscopic ellipsometry including recent efforts devoted to spectroscopic ellipsometry, mainly for 2D materials (*e.g.*, Figure 1b). In Section 2, we introduce the optical description of low-dimensional materials and their relation to the electronic structure of the material. In Section 3, we review three different ellipsometry techniques for 2D materials and each technique has its own advantage and disadvantage: (i) reflection contrast spectroscopy, (ii) conventional spectroscopic ellipsometry, and (iii) deterministic ellipsometry. An appropriate ellipsometry technique should be chosen based on the measurement characteristics. In Section 4, we discuss the technical challenges, practical problems, and future directions of ellipsometry for low-dimensional material studies. We believe that this review will benefit condensed matter physicists and material scientists trying to discover new low-dimensional materials, as well as nanophotonic scientists and engineers investigating the application of low-dimensional materials to optoelectronic devices.

## 2 Optical permittivity and spectroscopic ellipsometry

### 2.1 Optical permittivity as a fingerprint of material electronic structures

Many-body interactions in a lattice determine the local movement of electrons in a crystalline material. Owing to these interactions, the energy of the electrons is constrained to the electronic band structures *E*(**q**) according to the momentum **q** of electrons in the material. The characterization and analysis of the electronic structure of the material provide important information about its electric, magnetic, and optical properties. Many techniques, such as tunneling spectroscopy and angle-resolved photoemission spectroscopy (ARPES), have been suggested for analyzing the electronic structures of materials. In particular, optical spectroscopic ellipsometry, which generally does not require specialized setups, enables the rapid, robust, and noninvasive characterization of newly discovered materials regarding the current response related to the material electronic structure *E*(**q**) although it cannot provide direct information of *E*(**q**).

Spectroscopic ellipsometry measures the current response **J**(**q**, *ω*), that is, the expectation value of the quantum electrodynamic movement of electrons constrained by the material electronic structure, to an applied electric field **E**(**q**, *ω*). If the material of interest is linear and dielectric, the optical conductivity *σ*(**q**, *ω*) = **J**(**q**, *ω*)/**E**(**q**, *ω*) describes the material properties and some indirect but useful information of the material electronic structures: *e.g.*, the quasiparticle transition energies, the number of charge carriers within a finite frequency range, and the materials’ plasmonic response. In particular, quantum electrodynamic calculations provide a temperature-dependent expression of the optical conductivity [7],

(1) $$\sigma \left(q,\omega \right)=2i\omega \sum_{n,m\neq n}\frac{\Omega {\left|j_{q}^{nm}\right|}^{2}}{\omega_{mn}}\frac{e^{-E_{n}/k_{\text{B}}T}}{Z}\frac{1}{\omega \left(\omega +i\eta \right)-\omega_{mn}^{2}}\text{,}$$

for the interacting electrons in a lattice. The parameters Ω, *k*_B_, *Z*, and *η* are the unit volume, Boltzmann constant, partition function, and linewidth, respectively. The optical conductivity comprises the electronic transitions from the ground state |*n*〉 of energy *E*_*n*_ to the excited many-body state |*m*〉 of energy *E*_*m*_ at the transition energy *ω*_*mn*_ = *E*_*m*_ − *E*_*n*_. $j_{q}^{nm}=〈n|{\hat{j}}_{q}^{r}|m〉$ is the matrix element of the current operators. Therefore, when *σ*(**q**, *ω*) of a material is determined, information about the material electronic structures, such as the transition energies, linewidths, and temperature dependence, may be obtained.

In particular, optical techniques, including spectroscopic ellipsometry, measure the long-wavelength limit of the optical conductivity, $\lim_{q\to 0}\sigma \left(q,\omega \right)\equiv \sigma \left(\omega \right)$ because the photon energy is usually much smaller than 1 keV, which results in negligible optical momentum transfer **q** [7]. Although the zero-momentum limit is a drawback of spectroscopic ellipsometry, useful information on the electronic structures of the material may be obtained from *σ*(*ω*). In addition, the optical conductivity can be converted into other equivalent quantities such as the optical permittivity *ε*(*ω*) = 1 + 4πi*σ*(*ω*)/*ω* (in the Planck units) and refractive index $\tilde{n}\left(\omega \right)=\sqrt{\varepsilon \left(\omega \right)}$ for convenience for specific purposes. In spectroscopic ellipsometry, the optical permittivity *ε*(*ω*) is more commonly used than *σ*(*ω*).

**Figure 2:**
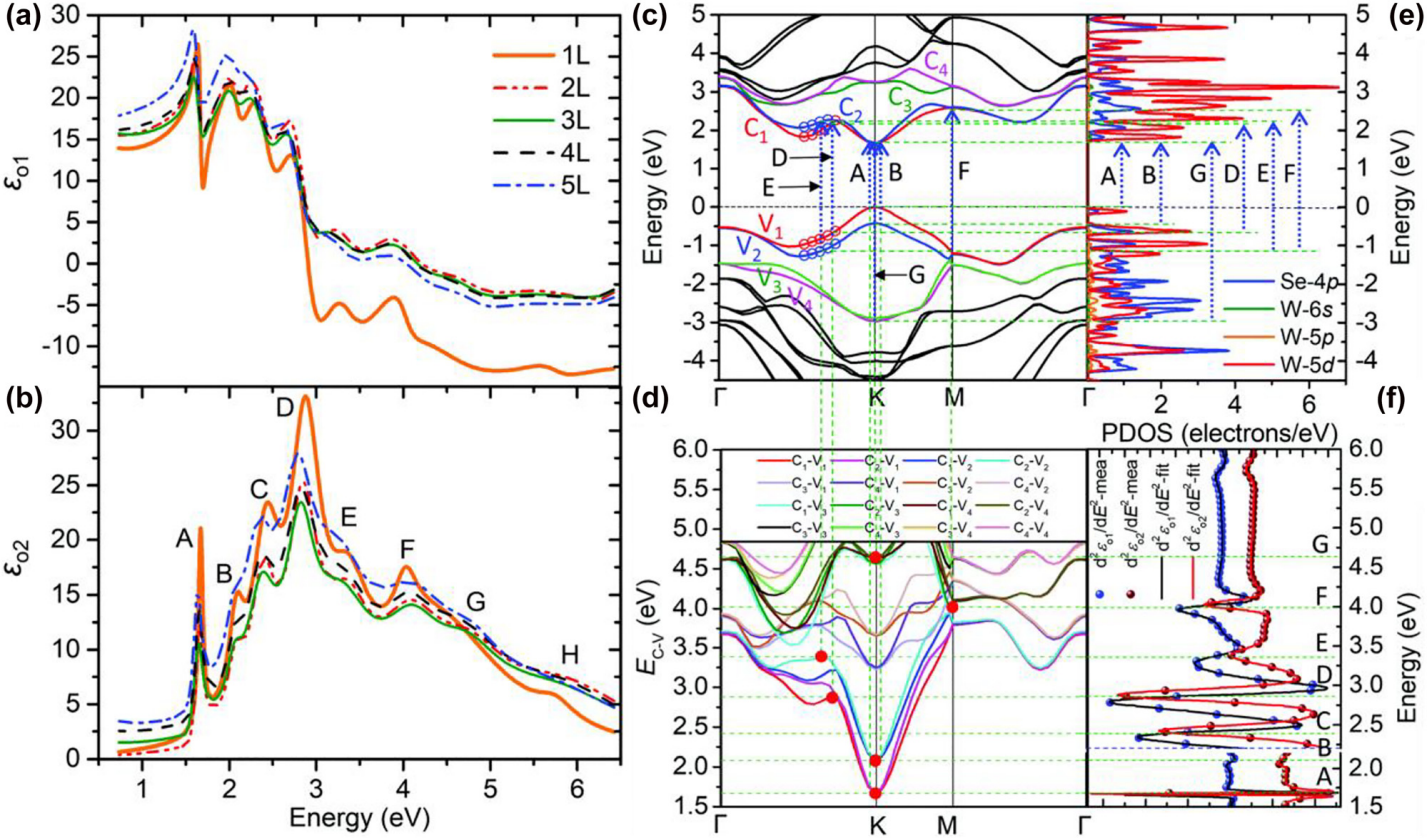
Optical permittivity and electronic band structure. (a) Real parts (*ε*_o1_) and (b) imaginary parts (*ε*_o2_) of the in-plane permittivity of 1–5L WSe_2_. (c) Corresponding electronic band structure, (d) energy differences *E*_C–V_ between the first four conduction and valence bands, (e) projected density of states (PDOS), and (f) critical point analysis of monolayer WSe_2_. Reprinted with permission from Ref. [8], copyright 2019, The Royal Society of Chemistry.

Figure 2 shows an example of the optical permittivity obtained using spectroscopic ellipsometry and its relation to the material electronic structures. Figure 2 compares the experimentally obtained permittivity spectrum (Figure 2a and b) to the first-principle calculations for the electronic structures (Figure 2c–f) of monolayer WSe_2_ [8]. In Figure 2b, the imaginary parts of the in-plane permittivity of 1–5 layers (L) WSe_2_ have eight peaks labeled from A–H. The real parts in Figure 2a exhibit Lorentzian lineshapes at the corresponding positions of *ε*_o2_ peaks. The first-principle calculations for the band structure (Figure 2c and d), projected density of states (PDOS, Figure 2e), and critical point analysis (Figure 2f) are consistent with the permittivity features [8]. Comparing the eight peaks (A–H) in Figure 2b to the band structure in Figure 2c, we conclude that the peaks correspond to the quasiparticle transitions in WSe_2_. The layer-dependent broadening of the permittivity in Figure 2a and b also demonstrates the effect of the interlayer interaction on each transition.

As shown in Figure 2, we can obtain important information on the material electronic structures. For example, we can extract the energies of quasiparticle transitions from the peaks of the imaginary part of the permittivity (Im{*ε*(*ω*)}). We may also estimate the exciton binding energy and spin–orbit splitting energy from permittivity measurements [9]. In general, other properties of materials at frequency *ω* can be determined. (i) Quasiparticle transitions occur if peaks of Im{*ε*(*ω*)} appear. (ii) The oscillator strength is proportional to the Im{*ε*(*ω*)} peaks. (iii) The material is metallic if *ε*(*ω*) < 0. (iv) Surface plasmons occur at the frequency *ω* satisfying *ε*(*ω*) = −1 at an air/metal interface. (v) The optical permittivity also provides information on the free-carrier density within a finite frequency range. The total number of charge carriers oscillating at different frequencies is conserved as follows:

(2) $$\text{Re}\int_{0}^{\infty }\sigma \left(\omega \right)\text{d}\omega =\frac{\pi n_{\text{e}}e^{2}}{2m_{\text{e}}}\text{,}$$

where *n*_e_, *e*, and *m*_e_ are the free charge carrier density, elementary charge, and electron mass, respectively. Equation (2) is called the *f*-sum rule or the Thomas–Reich–Kuhn rule [7]. and it demonstrates charge conservation in the material. It states that the integration over a finite range, *ω*_1_ < *ω* < *ω*_2_, $\text{Re}\int_{\omega_{1}}^{\omega_{2}}\sigma \left(\omega \right)\text{d}\omega$ , is proportional to the number of free charge carriers within the range. Recently, the f-sum rule was applied in the discovery of correlated plasmons in low-dimensional quantum materials such as monolayer Bi_2_Se_3_ and bulk WSe_2_ [10, 11].

### 2.2 Optical description of low-dimensional materials

**Figure 3:**
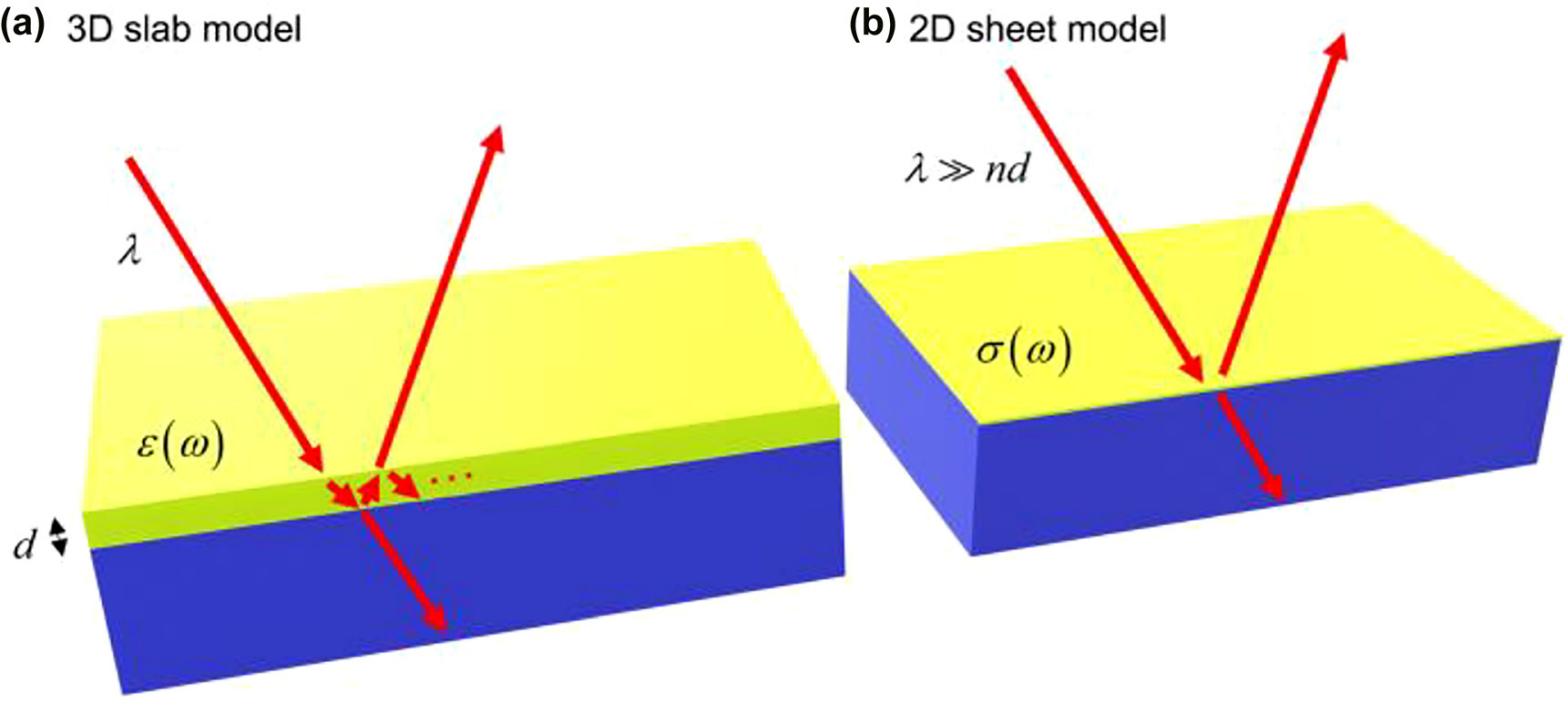
Two models for 2D monolayers. (a) 3D slab model with the bulk permittivity *ε*(*ω*) and finite thickness *d*, and (b) 2D sheet model with the sheet conductivity *σ*(*ω*) for a 2D monolayer (green) supported by a substrate (blue). In the 3D slab model, light (wavelength *λ*, red arrows) undergoes multiple reflections inside the monolayer. This does not occur in the 2D sheet model with no thickness. The 2D sheet model works well only for *λ* ≫ *nd* = √*εd*.

The linear optical response of 2D materials can be described by classical optics in two different but consistent ways. The first method models a 2D material as a homogenous three-dimensional (3D) slab of permittivity *ε*(*ω*) and finite thickness *d* (Figure 3a). The thickness (*d*) of the 2D material is usually chosen to be the interlayer spacing of the corresponding 3D bulk materials, for example, ∼0.6 and 0.34 nm for 2D transition-metal dichalcogenides (TMDs) and graphene, respectively [12]. The thickness *d* measured experimentally by atomic force microscopy (AFM) may also be chosen. The 3D slab model is generally applicable to 2D monolayers and heterostructures comprising multiple 2D layers.

In the other method, the 2D material is modeled as a dimensionless 2D conducting sheet possessing sheet conductivity *σ*(*ω*) (Figure 3b). The 2D sheet model is suitable for 2D monolayer and few-layered 2D materials where the optical phase shift of a single pass *φ* is much smaller than the unity, *i.e.*, |*φ*| = 2*π*|*n*|*d*/*λ* ≪ 1, where $\tilde{n}$ = √*ε* is the complex refractive index of the 3D slab model and *λ* is the wavelength of light in vacuum [13]. In general, the thickness of 2D materials is smaller than 1 nm, while the refractive index *n* does not exceed 10 in the visible spectral range of 400 nm < *λ* < 800 nm. Although the 2D sheet model is applicable only when the condition |*φ*| ≪ 1 is satisfied, it provides analytic advantage over the 3D slab model. In the 2D sheet model, one can deal with a single interface with dimensionless sheet conductivity *σ*(*ω*) to solve a reflection/transmission problem. Therefore, expressions of reflection and transmission are much simpler than the 3D slab model which has two interfaces, *i.e.*, air/slab and slab/substrate. Simple expression in the 2D sheet model allows us to extract information of physics analytically. For example, Dirac plasmons in graphene can be adequately described by the 2D sheet model [14], and they are consistent with experiments [15]. In addition, the photovoltaic Hall effect in graphene under the intense irradiation of circularly polarized light can be optically described by the 2D sheet model [16].

**Figure 4:**
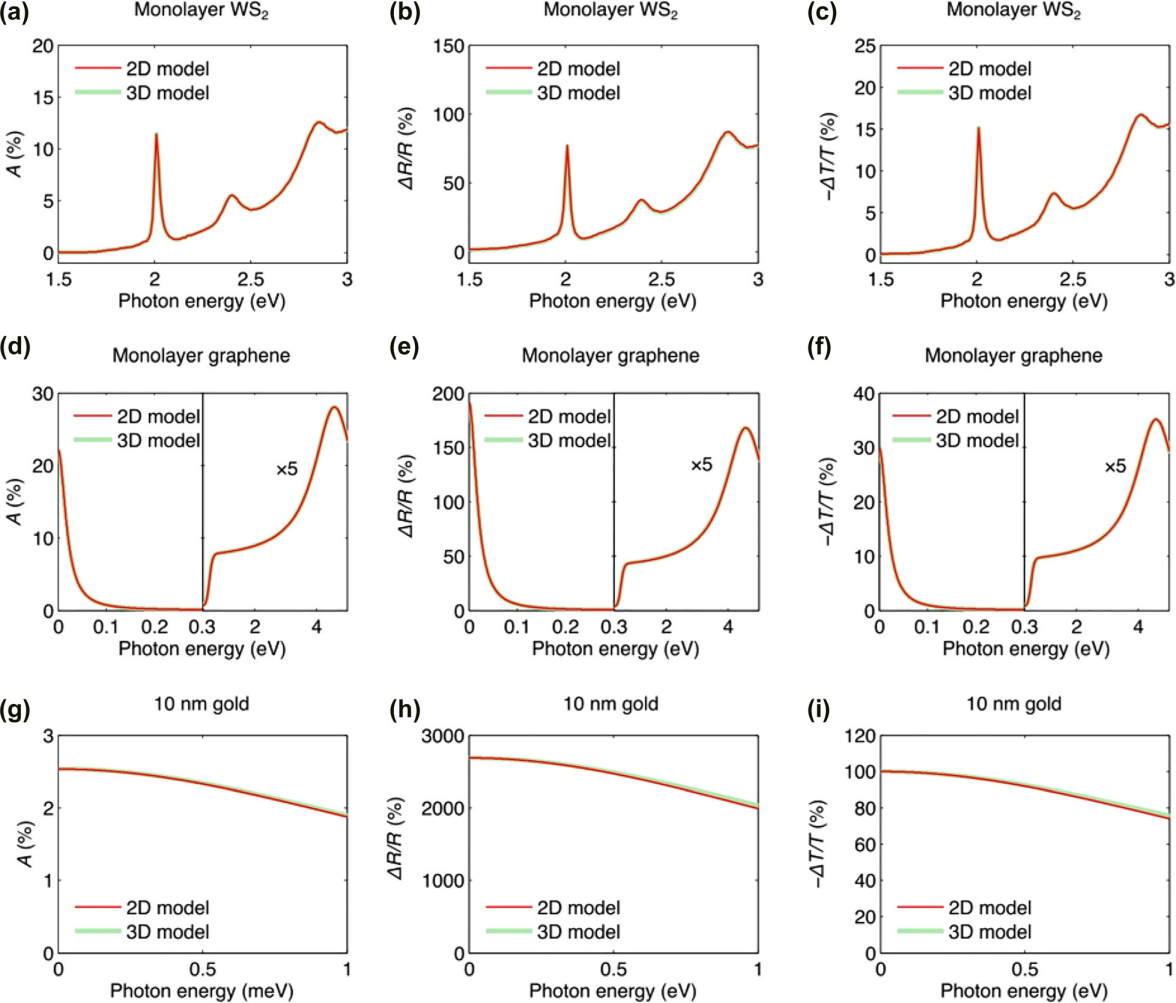
Absorption (*A*), reflection contrast (Δ*R*/*R*), and transmission contrast (Δ*T*/*T*) of (a–c) monolayer WS_2_, (d–f) graphene, and (g–i) 10 nm gold film on fused silica substrates. Reprinted with permission from Ref. [13], copyright 2018, IOP Publishing.

Both the 2D sheet and 3D slab models are consistent with each other when the condition |*φ*| ≪ 1 is satisfied; Figure 4 shows the absorption (*A*), reflection contrast (Δ*R*/*R*), and transmission contrast (Δ*T*/*T*) of 2D materials (WS_2_ and graphene) and 10 nm-thick gold film supported by fused silica substrates [13]. Both the 3D and 2D models exhibit consistent results in the visible and infrared spectral regions in Figure 3 because 2D materials and 10 nm-thick gold film exhibit |*φ*| < 5% and |*φ*| < 50%, respectively. Further, it has been reported that, even though one made a nano-patch antenna using graphene, both models are still consistent [17]. For 2D systems comprising multiple layers, both models can be used, but the 2D sheet model is applicable only when the optical phase shift amplitude |*φ*| of a single pass is much smaller than unity.

In this section, we have discussed the 3D and 2D models for 2D materials. Correspondingly, 1D materials such as carbon nanotubes can be described using two models: (1) a 3D cylinder model with permittivity *ε*(*ω*) and finite radius *R*, and (2) dimensionless 1D line model with the line conductivity *σ*(*ω*). Although the optical description of 1D materials has not been studied intensively, the 3D cylinder model was used to describe a Luttinger liquid plasmon in metallic single-walled carbon nanotubes at mid-infrared frequencies (details are provided in Section 4.3) [18, 19].

### 2.3 Spectroscopic ellipsometry

The reflection coefficients *r*^(s)^ and *r*^(p)^ of s- and p-polarized light, respectively, which incident obliquely on the sample surface, are different. We consider a sample consisting of *n* layers, where each layer has a complex-valued permittivity *ε*_*n*_(*ω*) and thickness *d*_*n*_. Spectroscopic ellipsometry measures the complex reflection coefficient ratio of s- and p-polarized light, *ρ* ≡ *r*^(p)^/*r*^(s)^ = tan*ψe*^iΔ^, where *ψ* and Δ are the changes in the amplitude ratio and phase, respectively [20]. On the other hand, we know the analytical form of the ratio *ρ* using a transfer matrix method [21]. Therefore, we obtain the *fundamental equation of ellipsometry* [20]

(3) $$\rho \left(\varepsilon_{n},d_{n}\right)=\text{tan}\;\psi \left(\theta \right)e^{i\Delta \left(\theta \right)}\text{,}$$

where the left- and right-hand sides of Eq. (3) correspond to the theoretically-known *ρ*(*ε*_*n*_, *d*_*n*_) and experimentally measured changes in the amplitude ratio (*ψ*) and phase (Δ) at a finite angle of incidence *θ*, respectively. Solving Eq. (3), we can determine *ε*_*n*_(*ω*) = Re*ε*_*n*_(*ω*) + *i*Im*ε*_*n*_(*ω*) and the thickness *d*_*n*_ of each layer of the sample. Because each layer has three parameters (Re*ε*_*n*_(*ω*), Im*ε*_*n*_(*ω*), and *d*_*n*_), the number of unknowns is 3*n*. The amount of experimental information required is larger than i*n*, while a single measurement at *θ* yields two parameters, *ψ* and Δ. Performing *m* experiments by varying the angle of incidence *θ* provides 2*m* of experimental information, making 2*m* > 3*n*. If the thickness of each layer (*d*_*n*_) is known using AFM measurements or assuming the interlayer spacing of the bulk material, the number of experiments *m* to obtain Re*ε*_*n*_(*ω*) and Im*ε*_*n*_(*ω*) is the same as the number of layers *n*.

**Figure 5:**
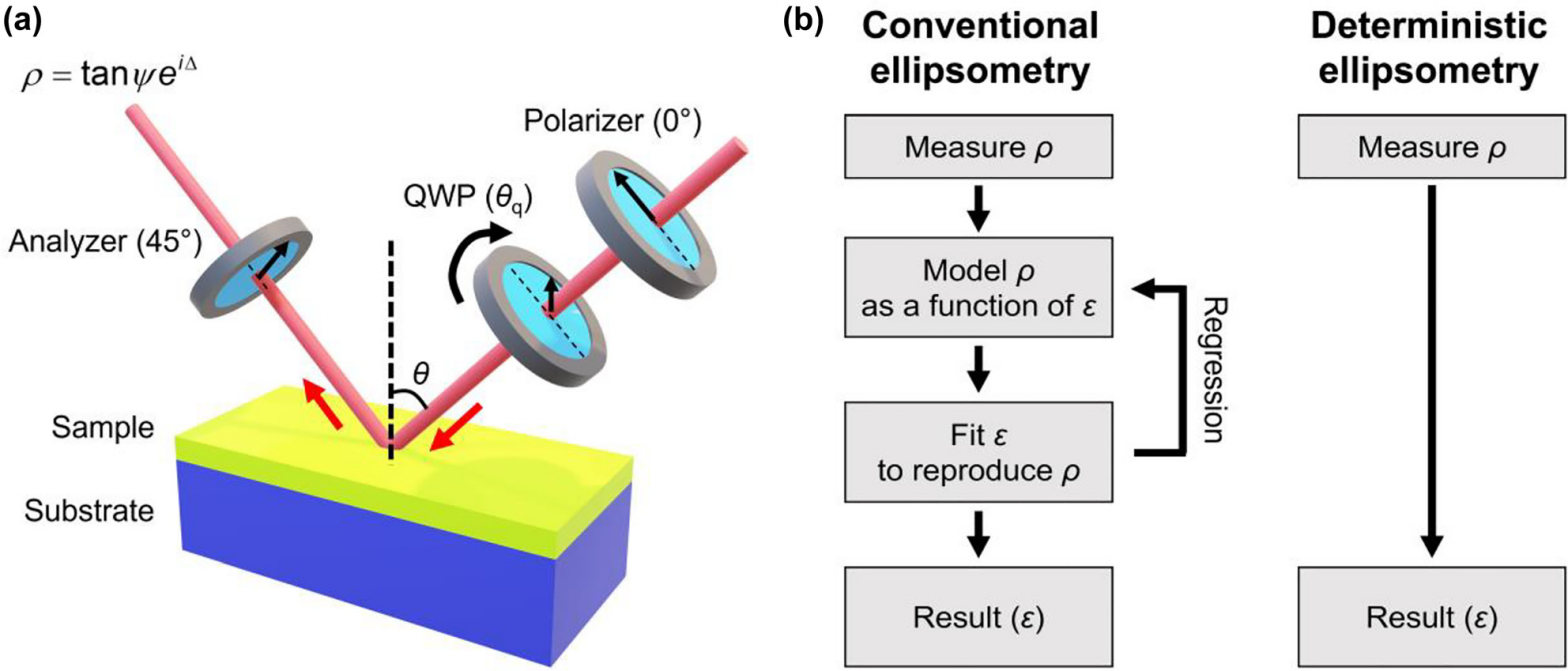
Experimental implementation of ellipsometry. (a) Schematic of a typical ellipsometry implementation called rotating compensator ellipsometry (RCE). Red arrows show the beam direction. QWP denotes the quarter wave plate. (b) Flowchart for conventional (left) and deterministic ellipsometry (right).

Many ellipsometry techniques have been suggested to measure the complex ratio *ρ* = tan *ψe*^iΔ^. Typical technique is rotating compensator ellipsometry (RCE), composed of a rotating wave plate and two fixed polarizers as shown in Figure 5a. The polarizer and the analyzer angles are fixed to 0° and 45° and angle of the wave plate varies as *θ*_q_. Then, the reflection intensity measured is proportional to [22].

(4) $$I\left(\theta_{\text{q}}\right)\propto 1-\frac{1}{2}N+S\;\text{sin}\left(2\theta_{\text{q}}\right)-\frac{1}{2}N\;\text{cos}\left(4\theta_{\text{q}}\right)+\frac{1}{2}C\;\text{sin}\left(4\theta_{\text{q}}\right)\text{,}$$

if the quarter wave plate (QWP) is used. Values *N*, *C*, and *S* are related to *ψ* and Δ by the relations, *N* = cos(2*ψ*), *C* = sin(2*ψ*)cos(Δ), and *S* = sin(2*ψ*)sin(Δ).

## 3 Spectroscopic ellipsometry for 2D materials

### 3.1 Reflection contrast spectroscopy for 2D materials

The simplest spectroscopic ellipsometry for 2D materials involves measuring the reflection contrast *δ*_R_ ≡ {*R*_s_(*ω*) − *R*_sub_(*ω*)}/*R*_sub_(*ω*) between a 2D sample *R*_s_(*ω*) and substrate *R*_sub_(*ω*) under light at normal incidence. We consider a 2D material described by a 3D homogeneous slab of permittivity *ε*(*ω*) and thickness *d*. The substrate is an infinite half-space dielectric with a purely real-valued refractive index, *n*_sub_. Because the wavelength of light *λ* = 2π/*k*_0_ is much larger than the atomic-level thickness *d*, the optical path length, *k*_0_*d*, is much smaller than unity. Therefore, expanding *δ*_R_ in the leading order of *k*_0_*d* yields (see Supplementary Materials for derivation)

(5) $$\delta_{\text{R}}\left(\omega \right)\equiv \frac{R_{\text{s}}\left(\omega \right)-R_{\text{sub}}\left(\omega \right)}{R_{\text{sub}}\left(\omega \right)}=\frac{4k_{0}d}{n_{\text{sub}}^{2}-1}\text{Im}\left\{\varepsilon \left(\omega \right)\right\}\text{.}$$

The analytical result, Eq. (5), shows that the reflection contrast spectrum *δ*_R_(*ω*) directly yields the imaginary part of the permittivity of the 2D material sample, Im{*ε*(*ω*)}, which is responsible for the absorption of light. Once Im{*ε*(*ω*)} is obtained within a spectral region of interest, the real part of the permittivity Re{*ε*(*ω*)} can be obtained numerically using the Kramers–Kronig relation:

(6) Re{ε(ω)/ε0}=1+1πP∫−∞∞Im{ε(ω′)/ε0}ω′−ωdω′,

where *P* is the principal value. The Kramers–Kronig relation (Eq. (6)) is a direct consequence of causality in the electromagnetic field [23]. Reflection contrast spectroscopy was first suggested to measure the graphene absorbance *πα* = 2.293% with the fine-structure constant *α* at visible frequencies [24]. After the experimental discovery of 2D TMDs, the reflection contrast *δ*_R_ became essential measure for optically characterizing excitonic features in 2D materials [13, 25], [26], [27].

The experimental implementation of reflection contrast spectroscopy is straightforward. 2D materials are prepared on highly reflective (large *R*_sub_) and nonabsorptive (purely real-valued *n*_sub_) substrates, for example, a Si substrate for visible frequencies, to ensure a low noise level and applicability of Eq. (5), respectively. *R*_s_ and *R*_sub_ can be measured separately to obtain *δ*_R_ using Eq. (5). *R*_s_ and *R*_sub_ are limited by shot noise, and thus, one can increase the light intensity or perform multiple measurements to suppress noise in *δ*_R_. Another advantage of reflection contrast spectroscopy based on Eq. (5) is a deterministic measurement that does not require *a priori* knowledge of the material electronic structures to determine Im{*ε*(*ω*)}.

Reflection contrast spectroscopy, however, has certain limitations: (i) The Kramers–Kronig relation can transform Im{*ε*(*ω*)} to Re{*ε*(*ω*)} to yield complete information of the complex-valued permittivity [23]. However, the transformation includes spectral integration from the DC limit (*ω* = 0) to infinity (*ω* → ∞). Spectroscopic measurements were always performed within a finite spectral region. Therefore, the truncated numerical integration in the Kramers–Kronig relation can introduce inaccuracies in Re{*ε*(*ω*)}. (ii) 2D materials are usually prepared on thick oxide spacers (to make atomically thin 2D materials visible), hBN flakes (to have an atomically flat substrate) or other 2D material layers (to construct heterostructures). These complicated structures can introduce interference of light, which renders Eq. (5) invalid and the measured *δ*_R_(*ω*) no longer indicates absorption by the 2D material, namely, Im{*ε*(*ω*)}.

### 3.2 Conventional spectroscopic ellipsometry for 2D materials

The conventional spectroscopic ellipsometry discussed in Section 2.3 has been actively used to measure the permittivity of 2D materials. Li et al. performed spectroscopic ellipsometry for monolayer MoS_2_, MoSe_2_, WS_2_, and WSe_2_ supported by sapphire substrates and determined the exciton binding energy and spin–orbit splitting [9]. Heinz et al. complemented the previous works by Kramers–Kronig-constrained analysis to obtain the permittivity of 2D TMDs [12]. Liu et al. conducted spectroscopic ellipsometry to study the layer-dependent optical permittivity from monolayer to few-layer WSe_2_ [8]. Volkov et al. reported broadband spectroscopic ellipsometry for monolayer and bulk MoS_2_ from 290 to 3300 nm [28]. Working with a commercial ellipsometer company, Islam et al. also reported the in-plane and out-of-plane permittivity of monolayer, few-layer, and thin-film MoS_2_ over a broad spectral range from 190–1700 nm while working with a commercial ellipsometer company [29].

Conventional spectroscopic ellipsometry aims at solving the fundamental equation of ellipsometry (Eq. (3)) using experimentally obtained quantities. Experimental procedure of conventional spectroscopic ellipsometry is summarized in the left panel of Figure 5b. Although Eq. (3) appears simple, the left-hand side term, *ρ*(*ε*_*n*_, *d*_*n*_), is usually complicated. There is no simple closed-form expression for the permittivity *ε* of the slab even for a single slab on a substrate [20]. Therefore, conventional spectroscopic ellipsometry usually assumes that the permittivity *ε*_*n*_(*ω*) of the sample follows specific lineshape functions to fit *ρ*(*ε*_*n*_, *d*_*n*_) in Eq. (3). For example, to obtain the permittivity of 2D TMDs, Ref. [12] assumes that their permittivity is composed of *N* Lorentzian oscillators as follows:

(7) $$\varepsilon \left(\omega \right)=1+\sum_{k=1}^{N}\frac{f_{k}}{\omega_{k}^{2}-\omega^{2}-i\omega \gamma_{k}}\text{,}$$

where *ω*_*k*_, *f*_*k*_, and *γ*_*k*_ are the quasiparticle transition frequency, oscillator strength, and linewidth of the *k*th transitions, respectively. Ref. [12] included transitions up to 30 eV for the spectral region of 1.5–3.0 eV because the high-energy transitions significantly influence the low-energy permittivity in 2D TMDs. Note that the Lorentzian expression for the permittivity, Eq. (7) can be derived from the quantum electrodynamic expression, Eq. (1) [7].

In addition, other types of oscillator lineshape functions can be used to obtain better fitting of *ρ*(*ε*_*n*_, *d*_*n*_), instead of the Lorentz oscillators in Eq. (7). Tauc–Lorentz (TL) and Cody–Lorentz (CL) oscillators are typical lineshape functions that modify and complement Lorentz oscillators. In the TL and CL oscillator models, the Tauc and Cody lineshape functions, which describe the absorption process in semiconductors, are multiplied by the imaginary part of the Lorentz oscillator permittivity (Eq. (7)), respectively [30]. Subsequently, the real part of the permittivity is obtained by the Kramers–Kronig relation. For example, spectroscopic ellipsometry for monolayer and few-layer WSe_2_ (Figure 2a and b) used three CL oscillators and five Lorentz oscillators for the in-plane permittivity, while it used a single TL oscillator for the out-of-plane permittivity in the spectral range of 0.73–6.42 eV [8]. The choice of the oscillator lineshape function in the fitting procedure depends on the characteristics of quasiparticle transitions. These fitting oscillator functions enables conventional spectroscopic ellipsometry to perform regression analysis to minimize the mean-squared-error (MSE), characterizing the difference between the fitting-generated reflection spectrum (*ρ*(*ε*_*n*_, *d*_*n*_)) and the experimentally-obtained ground truth spectrum [20, 22].

The application of conventional spectroscopic ellipsometry to 2D materials is the most general approach that does not require specific conditions, as in reflection contrast spectroscopy (Section 3.1). If other techniques fail owing to experimental situations, one should return to conventional spectroscopic ellipsometry. In addition, the oscillator lineshape functions, such as the Lorentzian (Eq. (7)) satisfy the Kramers–Kronig relation. Using Kramer–Kronig-constrained lineshape functions to fit the reflection spectrum, the causal permittivity of the permittivity for a given material sample can be obtained.

### 3.3 Deterministic ellipsometry for 2D materials

**Figure 6:**
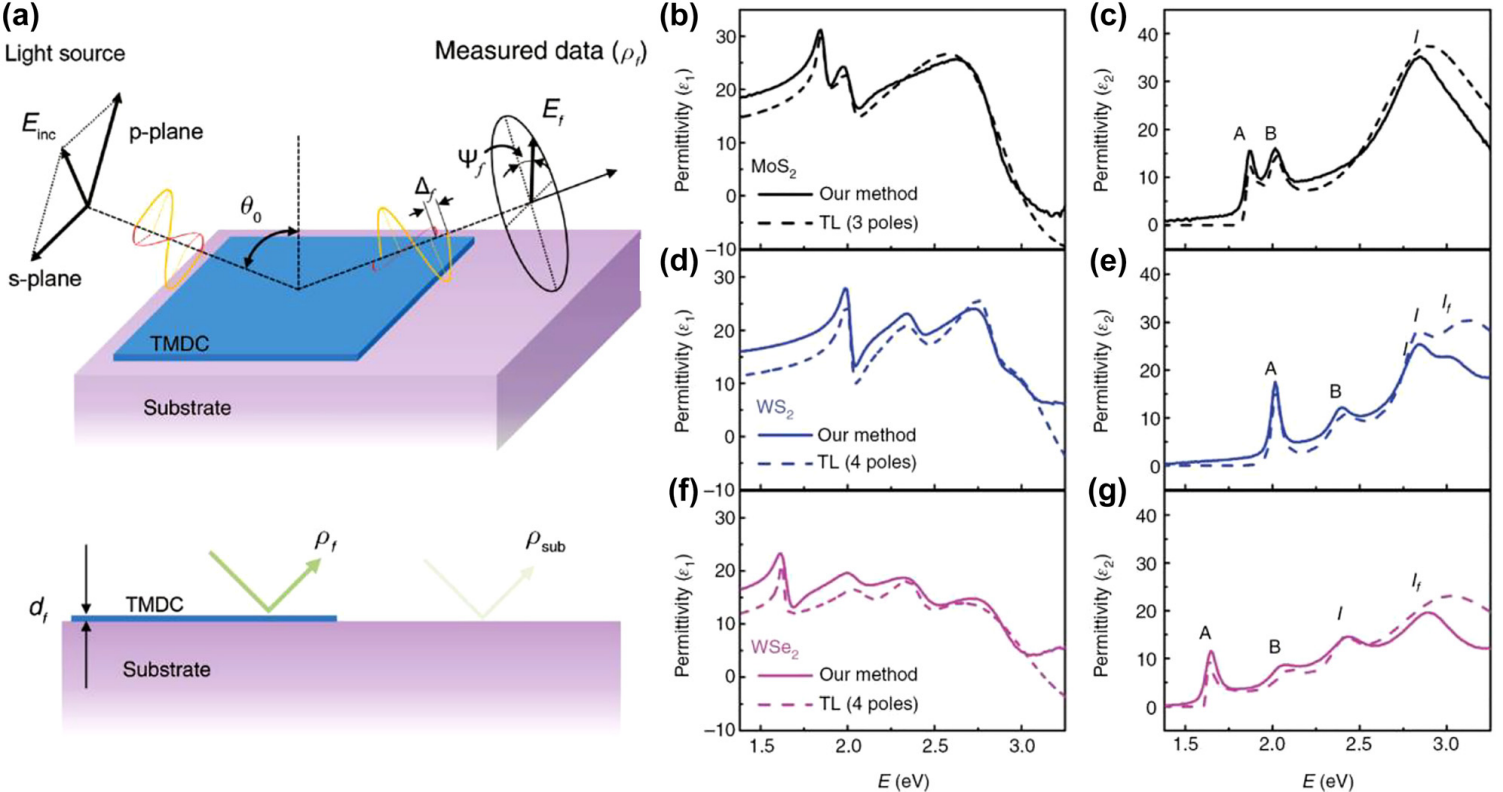
Deterministic ellipsometry for 2D materials. (a) Schematic of the deterministic ellipsometry for 2D materials. Comparison of the permittivity *ε* = *ε*_1_ + *iε*_2_ obtained by the deterministic ellipsometry (solid lines) and conventional spectroscopic ellipsometry with few TL oscillators (dashed lines) for (b and c) monolayer MoS_2_, (d and e) WS_2_, and (f and g) WSe_2_. Reprinted with permission from Ref. [30], copyright 2018, de Gruyter.

As discussed, the reflection contrast spectroscopy (Section 3.1) measures the imaginary part of the permittivity, Im{*ε*(*ω*)}, alone, and the numerically converted Re{*ε*(*ω*)} is inaccurate if the spectral window is narrow. Although conventional spectroscopic ellipsometry is powerful, it depends on fitting spectral lineshape functions that require *a priori* knowledge of material electronic structures; this is because high-energy transitions can significantly affect lower energy permittivity. The lack of detailed information of material electronic structures is a huge drawback in studies of newly discovered 2D materials [31]. We proposed deterministic ellipsometry for 2D materials to overcome the limitations of both techniques. Experimental procedure of deterministic ellipsometry is summarized in the right panel of Figure 5b. Importantly, it does not require regression analysis for model of *ρ* (Figure 5b). As illustrated in Figure 6a, we combined two techniques to overcome the limitations of each. First, we measure the *ρ* contrast defined by *δ*_*ρ*_(*ω*) ≡ {*ρ*_s_(*ω*) − *ρ*_sub_(*ω*)}/*ρ*_sub_(*ω*) in the analogy of the reflection contrast spectroscopy. The subscripts ‘s’ and ‘sub’ in *ρ* denote the reflection coefficient ratios of the 2D sample and bare substrate. As in the case of conventional spectroscopic ellipsometry (Section 2.3), the complex-valued *δ*_*ρ*_(*ω*) provides two solvable equations for two unknowns Re{*ε*(*ω*)} and Im{*ε*(*ω*)}. Because 2D materials have an atomic thickness, the analytic expression for *δ*_*ρ*_(*ω*) can be expanded in the leading order of *k*_0_*d*, yielding a simple expression for the permittivity of 2D materials on a nonabsorptive substrate (purely real-valued *n*_sub_),

(8) $$\varepsilon \left(\omega \right)=\frac{1}{2}\left[1+n_{\text{sub}}^{2}+\left\{\delta_{\rho }\left(\omega \right)/\alpha \left(\omega \right)\right\}\right]\pm \frac{1}{2}\sqrt{{\left[1+n_{\text{sub}}^{2}+\left\{\delta_{\rho }\left(\omega \right)/\alpha \left(\omega \right)\right\}\right]}^{2}-4n_{\text{sub}}^{2}}\text{,}$$

The sign in Eq. (8) is determined by the sample passivity condition, Im{*ε*(*ω*)} > 0. The function *α*(*ω*) contains information about the incident angle *θ* and 2D sample thickness *d* as follows:

(9) $$\alpha \left(\omega \right)=-4ik_{0}d\frac{n_{\text{sub}}^{2}\;\text{cos}\;\theta \;{\text{sin}}^{2}\;\theta }{\left(n_{\text{sub}}^{2}-1\right)\left\{\left(n_{\text{sub}}^{2}-1\right)+\left(n_{\text{sub}}^{2}+1\right)\text{cos}\left(2\theta \right)\right\}}\text{,}$$

where *c* is the speed of light. When *α*(*ω*) is known and *δ*_*ρ*_(*ω*) is measured, Eq. (8) provides a deterministic solution of the permittivity in the spectral region of interest. It requires neither the Kramers–Kronig transformation, as in the reflection contrast spectroscopy (Section 3.1) nor the fitting, as in the conventional spectroscopic ellipsometry (Section 3.2). It is to be noted that the leading order (*k*_0_*d*)-based method in Eq. (8), has also been extended to the second-order (*k*_0_*d*)^2^ expansion for the short-wavelength region or thick samples [32].

Figure 6b–g compare the deterministic ellipsometry results with the fitting results in the conventional spectroscopic ellipsometry if oscillators only in the spectral region of interest are included. The two ellipsometry techniques use the same complex reflection coefficient ratio *ρ* measured in the same sample; the deterministic ellipsometry (solid lines in Figure 6) uses Eq. (8), while the conventional ellipsometry (dashed lines in Figure 6) use three TL oscillators to fit the experimentally measured spectrum of *ρ*. In 2D TMDs, three or four excitonic features are shown at visible frequencies, but higher energy features outside the spectral range affect the visible spectrum significantly. This introduces inaccuracies in the conventional spectroscopic ellipsometry results in Figure 6. If newly discovered 2D materials or heterostructures are being considered, information regarding high-energy electronic structures may remain unknown. Owing to the energy limitation up to 3–5 eV in conventional spectroscopic ellipsometry, the absence of high-energy information results in ambiguity in the experimental determination of the permittivity.

The deterministic ellipsometry introduced in this section inherits the advantages of reflection contrast spectroscopy. It is straightforward and error-robust, and does not require *a priori* knowledge of the material electronic structure to determine the complex-valued permittivity. However, it is only applicable to a 2D monolayer on a nonabsorptive substrate, as discussed in Section 3.1. Both reflection contrast spectroscopy and deterministic ellipsometry fail in characterizing the permittivity of multiple layers in a heterostructure, making it necessary to revert to conventional spectroscopy. This limitation is discussed in detail in Section 4.2.

### 3.4 Other techniques

**Figure 7:**
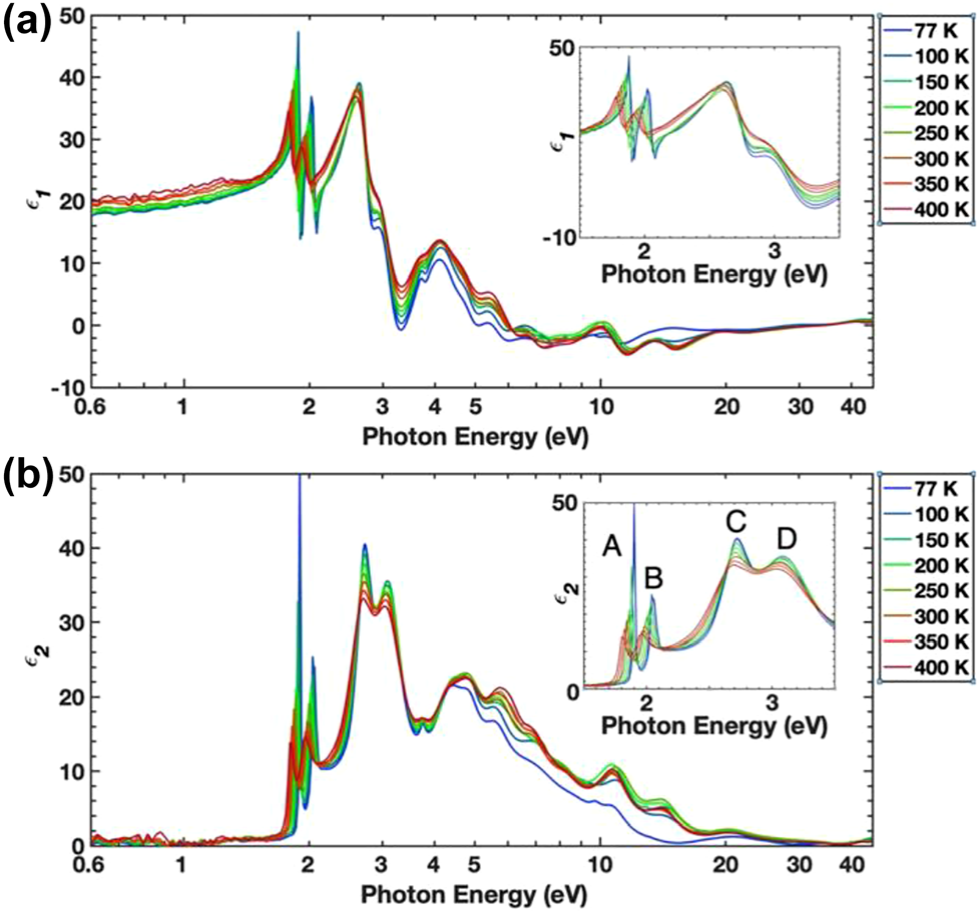
Ultrabroad band spectroscopic ellipsometry using synchrotron radiation. (a) Real parts (*ε*_1_) and (b) imaginary parts (*ε*_2_) of the permittivity of bulk MoS_2_ at various temperatures. Reprinted with permission from Ref. [11], copyright 2021, Nature Publishing Group.

Although it is desirable to obtain the permittivity within visible frequencies from 1.5 to 3 eV using spectroscopic ellipsometry, the effect of electronic transitions up to 30 eV need to be included [12]. Conventional spectroscopic ellipsometry usually includes photon energies up to 3.0–5.5 eV. This mismatch in the energy scale yields inaccurate measurements. Recently, synchrotron radiation with a high-intensity photon beam of energy up to 45 eV has been applied to measure the optical permittivity of bulk MoS_2_ [11]. Figure 7 shows the effect of temperature on the permittivity of bulk MoS_2_ measured using synchrotron radiation. In Figure 7, A–D excitons are clearly visible below 3.4 eV, while four highly temperature-dependent high-energy peaks appear at ∼5, ∼6, ∼11, and ∼15 eV. Using permittivity measurements, this ultrabroadband high-energy spectroscopic ellipsometry reveals high-energy bands associated with the *p*-*d* hybridizations as well as the existence of unconventional soft X-ray-correlated plasmons occurring in a material with a low free charge density.

**Figure 8:**
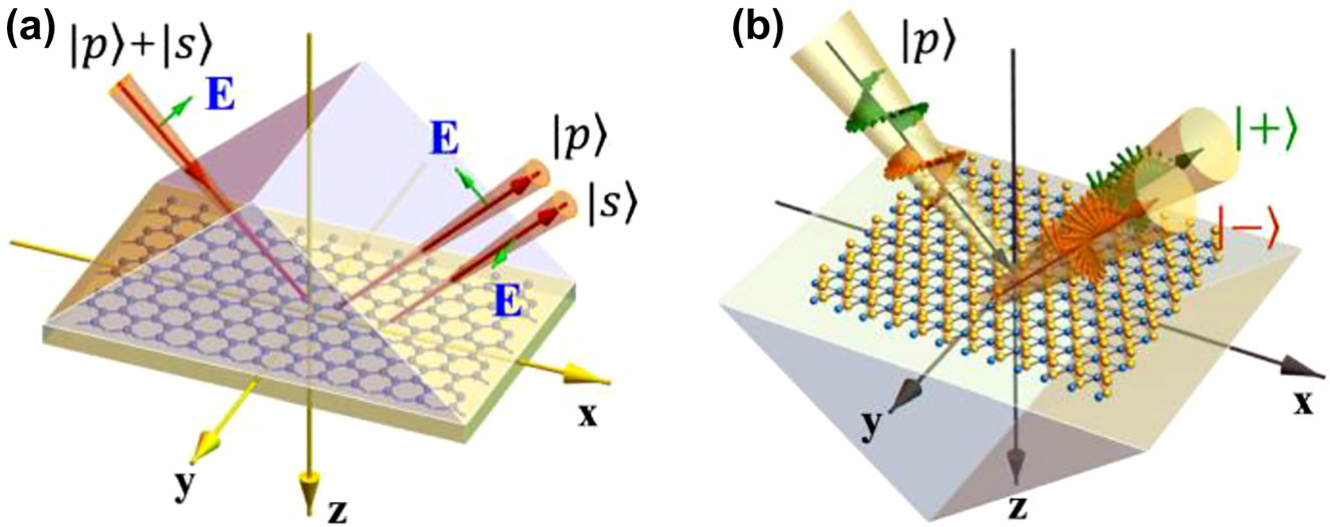
Alternative technique to obtain the optical permittivities of 2D materials. (a) Goos–Hӓnchen shift and (b) photonic spin-Hall effect of a 2D monolayer supported by a prism. Reprinted with permission from Ref. [32], copyright 2021, IOP Publishing Ltd.

All the techniques introduced above utilize ellipsometry, which measures the changes in the amplitude ratio *ψ* and phase Δ of the reflection coefficient ratio of s- and p-polarized light. Recently, alternative approaches for measuring the optical permittivity of 2D materials have been reported [33]. When 2D materials are coated on a prism, the linearly polarized reflected beam is split into p- and s-polarized light by the Goos–Hӓnchen shift (Figure 8a) or left- and right-handed circularly polarized light by the photonic spin Hall effect (Figure 8b). Both shifts are sensitive to the optical parameters of 2D materials on a prism, and hence, they are applied to measure the optical conductivity [34] and number of layers of 2D materials [35, 36]. Combining the weak measurement techniques, these alternative measurements can provide a high resolution of the optical permittivity [33]. Another alternative approach uses a scattering-type scanning near-field optical microscopy (s-SNOM) to obtain the permittivity of 2D materials in 2D heterostructures [37]. Basic principle is similar with the conventional spectroscopy; s-SNOM measures scattering amplitude and phase. Then, theoretical model of the permittivity can be calculated by the Lorentzian oscillator with a point dipole model for the tip apex. Regression analysis provides fitting parameters reproducing the measured scattering amplitude and phase. Advantage of s-SNOM-based ellipsometry is high spatial resolution up to 20 nm.

## 4 Technical challenges and other issues

### 4.1 Interference effect in 2D heterostructures

Reflection contrast spectroscopy (Section 3.1) and its application to ellipsometry (Section 3.3) are based on a single pass of light through atomically thin layers. However, 2D material research often requires thick insulating layers to make 2D materials visible (*e.g.*, by SiO_2_ layer on Si substrate) or prepare atomically flat substrates (*e.g.*, hBN flakes). In addition, 2D materials can be stacked to form heterostructures that induce novel phenomena such as the moiré flat band [2, 27, 38], [39], [40], [41]. This breaks down the validity of the reflection-contrast-based ellipsometry because the reflected light starts to experience interference from multiple reflections inside the sample.

**Figure 9:**
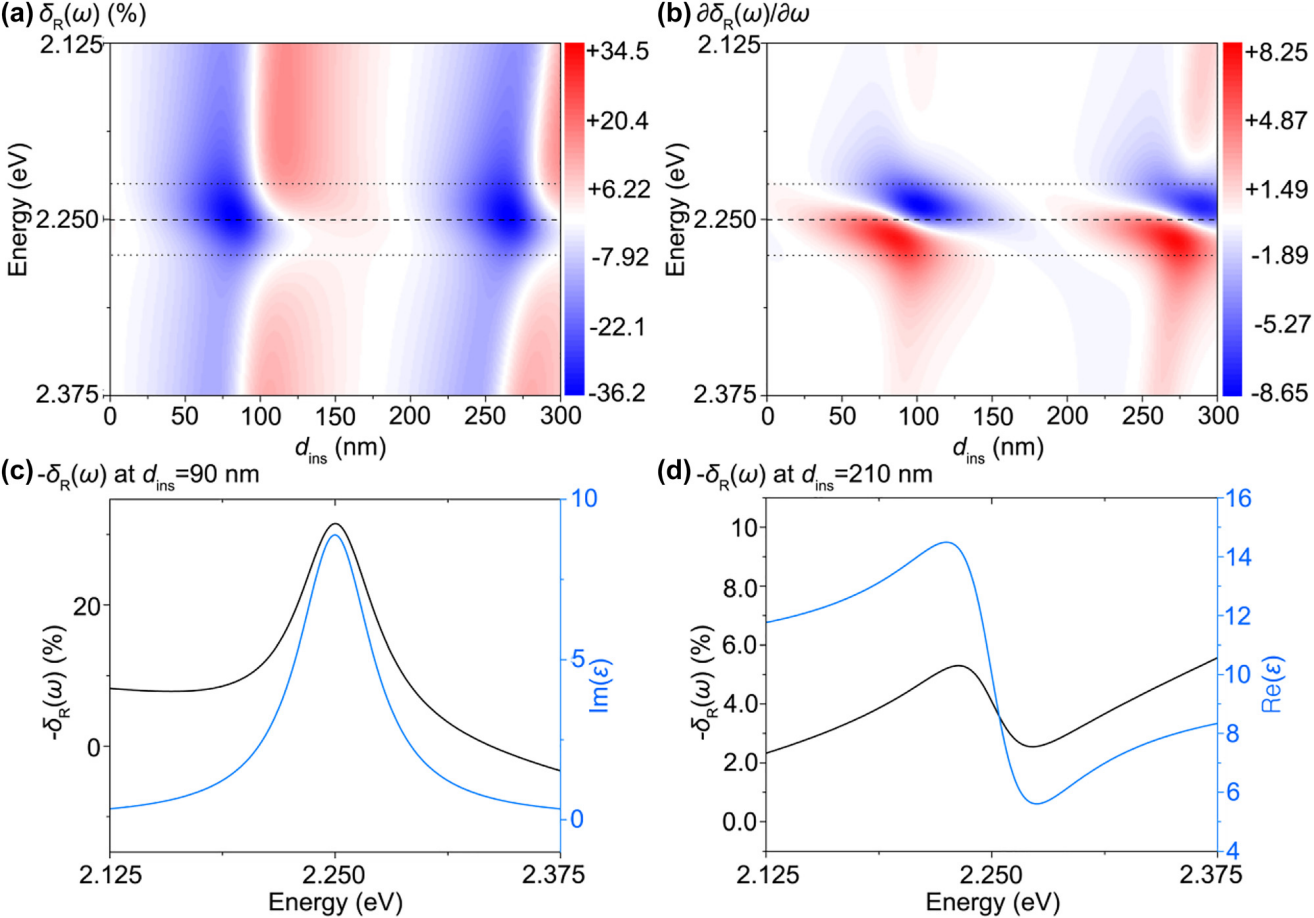
Limitation of reflection contrast spectroscopy. (a) Reflection contrast spectra *δ*_R_(*ω*) of a 2D material/insulator/Si substrate according to thickness of the insulating layer *d*_ins_. (b) Derivative of the reflection contrast spectra ∂*δ*_R_(*ω*)/∂*ω*. The dashed lines correspond to the transition energy (*ħω*_1_) of 2.25 eV, while the dotted lines correspond to the linewidths *ħ*(*ω*_1_ − *γ*_1_)/2 ≤ *ħω*_1_ ≤ *ħ*(*ω*_1_ + *γ*_1_)/2. Specific examples of the reflection contrast spectra *δ*_R_(*ω*) of a 2D material/insulator/Si substrate at (c) *d*_ins_ = 90 nm and (d) *d*_ins_ = 210 nm (black lines). The real and imaginary parts of the permittivity of the 2D material are plotted for comparison in Figure 7d and e (blue lines).

For example, Figure 9a shows the changes in the reflection contrast spectra *δ*_R_(*ω*) of 2D materials on an insulating layer (refractive index *n*_ins_ = 1.5, thickness *d*_ins_) supported by a Si substrate (*n*_sub_ = 4.0). Reflection was measured using normally incident light. As shown in Figure 9, 2D materials exhibit a single Lorentzian oscillator transition (Eq. (7)) whose transition frequency and linewidth are *ħω*_1_ = 2.25 eV and *ħγ*_1_ = 50 meV, respectively. Further, the background permittivity is 10, instead of 1, in Eq. (7). If 2D materials occur directly on the Si substrate without an insulating layer (*d*_ins_ = 0), the reflection contrast spectra *δ*_R_(*ω*) shows the imaginary part of the permittivity of the 2D material, Im{*ε*(*ω*)}, according to Eq. (5). As the insulator thickness *d*_ins_ increases, the features of the real and imaginary parts of the permittivity are mixed in *δ*_R_(*ω*), as shown in Figure 9a. Peaks and dips of the reflection contrast, *i.e.*, ∂*δ*_R_(*ω*)/∂*ω* = 0 (Figure 9b), mainly appear in the region *ħ*(*ω*_1_ − *γ*_1_)/2 ≤ *ħω*_1_ ≤ *ħ*(*ω*_1_ + *γ*_1_)/2, where both boundaries correspond to the peak and dips of the real part of the Lorentzian lineshape. To measure the features of Im{*ε*(*ω*)} in *δ*_R_(*ω*) (Figure 9c), only specific conditions such as *d*_ins_ ∼ 90 nm should be satisfied, whereas *δ*_R_(*ω*) generally mixes the features of Re{*ε*(*ω*)} and Im{*ε*(*ω*)} (Figure 9a). Likewise, other conditions such as *d*_ins_ ∼ 210 nm show the features of Re{*ε*(*ω*)} in *δ*_R_(*ω*) (Figure 9d). Therefore, we can conclude that the reflection contrast spectra for thick layered systems show mixing of the real and imaginary parts of the permittivity. In addition, the influence of the oxide/substrate reflection introduces the repeating patterns (blue and red regions), which cannot be removed in *δ*_R_(*ω*) (Figure 9a).

As shown in Figure 9, the reflection contrast measurement on a thick layer supported by a substrate cannot directly provide permittivity information. This interference effect causes the breakdown of reflection contrast spectroscopy (Section 3.1) and its application to deterministic ellipsometry (Section 3.3). It is necessary to revert to conventional spectroscopic ellipsometry discussed in Section 3.2 if the reflection contrast spectroscopy fails to yield the permittivity. We also note that one should be careful when assigning spectral features to the electronic origins of the materials in the reflection contrast spectrum *δ*_R_(*ω*) if the system of interest contains multiple or thick layers that introduce the interference of reflected light.

### 4.2 Beyond the linear isotropic regime: anisotropy, chirality, and nonreciprocity

We have focused on 2D materials whose linear, isotropic, dielectric, and nonmagnetic responses are optically described by the electromagnetic constitutive relations given by **D** = *ε***E** and **B** = *μ*_0_**H**, where **D**, **E**, **B**, **H**, and *μ*_0_ are the displacement field, electric field, auxiliary field, magnetic field, and vacuum permeability, respectively. However, this optical description is sometimes insufficient for describing the optical response of 2D materials because the constitutive relations can be nonlinear and anisotropic to the applied electric field in some cases. For example, (i) in general, the permittivity is a tensor and not a scalar, owing to the intrinsic anisotropy of the crystal structure. (ii) 2D materials can exhibit chiral and/or nonreciprocal responses with external perturbation. To the best of our knowledge, ellipsometric studies of 2D materials with complicated constitutive relations have rarely been performed. In addition, it is intriguing to determine whether the reflection contrast spectrum can deterministically provide all the optical parameters in the complicated constitutive relations. We briefly review two cases beyond the linear isotropic regime in the constitutive relations of 2D materials to promote future efforts for novel ellipsometry techniques.

First, 2D materials are anisotropic because their atomic crystals extend in the in-plane direction, while they are terminated along the out-of-plane direction, making them 2D. They can be optically described by the permittivity tensor of a uniaxial crystal, namely, $\varepsilon^{↔}$ = diag(*ε*_o_, *ε*_o_, *ε*_e_), where *ε*_o_ and *ε*_e_ denote the ordinary in-plane and extraordinary out-of-plane permittivities, respectively [8]. Fortunately, the anisotropy of 2D materials does not affect the reflection by a monolayer on a substrate [8, 29, 31]. It has been reported that the effect of anisotropy in TMD becomes significant when the thickness becomes a few tens of nanometers [29]. However, for complicated 2D heterostructures composed of multiple 2D layers and thick hBN flakes, anisotropy should be considered in spectroscopic ellipsometry because their thickness can be comparable to the wavelength of light. In addition, the out-of-plane permittivity may play an important role in nanophotonic applications, because resonant nanostructures and metamaterials can induce strong out-of-plane components of the electromagnetic fields [42], [43], [44].

Second, 2D materials can show chiral and/or nonreciprocal responses, originating from breaking the inversion and time-reversal symmetries, respectively. The inversion symmetry can be broken intrinsically or extrinsically. In the 2H phase of 2D TMDs, the lack of intrinsic inversion symmetry in their crystal structures enables the orbital magnetic moment accompanying optical circular dichroism (CD), which is different optical properties induced by left- or right-circularly polarized light [3, 4]. A potential difference across 2D materials is induced by applying electrostatic fields normal to 2D materials, resulting in broken inversion symmetry. In general, breaking the time-reversal symmetry requires external perturbations. High DC magnetic fields [45], the optical Stark effect without magnetic fields [46] the magnetic proximity effect [47], and the direct current application [48], [49], [50] can break the time-reversal symmetry. A time-reversal broken chiral superconducting phase was also predicted by electron correlations in a single trilayer TiSe_2_ [51]. When the inversion symmetry and/or the time-reversal symmetry are broken in 2D monolayers and heterostructures, the optical permittivity of linear dielectric materials is not sufficient to describe their chiroptical responses. Optical descriptions of chiral and/or nonreciprocal materials differ from those of dielectric materials; the constitutive relations become **D** = *ε***E** + (*χ* + *iκ*)**H**/*c*_0_ and **B** = *μ*_0_**H** + (*χ* − *iκ*)**E**/*c*_0_, where *χ* and *κ* are the nonreciprocity and chirality parameters, respectively [52]. The scalar optical parameters *ε*, *χ*, and *κ* also become tensors if the materials are anisotropic, as in 2D materials.

### 4.3 Permittivity of 1D materials

This review mainly discusses ellipsometry for 2D materials and heterostructures because the amplitude and phase information of light reflected by 2D materials can be obtained in a straightforward manner. This reflection-based approach for 2D materials cannot be applied to 1D materials whose radii are atomically small, such as carbon nanotubes, gold atom chains, mirror-twin boundaries in 2D TMDs, conducting polymers, and quantum Hall edges. For these 1D materials, alternative techniques are necessary for experimentally characterizing their optical response. Similar to 2D materials whose optical responses are described by the 3D slab model of permittivity *ε*(*ω*) and slab thickness *d*, 1D materials can be described by a 3D solid cylinder of finite radius *R* filled with permittivity *ε*(*ω*). As discussed briefly in Section 2.2, 1D materials can be described by a dimensionless 1D line current of the line conductivity *σ*(*ω*). A detailed analysis of the difference between the two models for 1D materials, namely, the 3D cylinder and 1D line models, is absent. However, the 3D cylinder model has been shown to describe the optical response of 1D metallic single-walled carbon nanotubes (SWNTs) that support a Luttinger liquid.

In brief, the permittivity of metallic SWNTs, *ε*_SWNT_(*ω*), can be experimentally determined by measuring the plasmon wavelength *λ*_p_, plasmon quality factor *Q*, and SWNT radius *R*. Metallic SWNTs have linear electronic dispersion up to near-infrared frequencies (∼1 eV) [53]; thus, they behave as a Luttinger liquid, a strongly correlated electronic matter in 1D metals. The optical and electronic responses of the Luttinger liquid are described by the Luttinger liquid interaction parameter *g*. It is determined by the experimentally observable *λ*_p_ and *R* using the expression [18, 54]:

(10) $$g=1/\sqrt{1+\frac{8e^{2}}{\pi \hbar v_{\text{F}}}K_{0}\left\{\left(2\pi /\lambda_{\text{p}}\right)R\right\}}\text{,}$$

where the Fermi velocity, reduced Plank constant, and zeroth-order modified Bessel function of the second kind are *v*_F_, *ħ*, and *K*_0_, respectively. Then, *g* yields the metallic SWNT permittivity in the approximate form (in SI units) [18],

(11) $$\text{Re}\left\{\varepsilon_{\text{SWNT}}\left(\omega \right)\right\}=-\frac{16}{1-g^{2}}\frac{k_{e}e^{2}}{\varepsilon_{\text{eff}}\hbar v_{\text{F}}}\frac{v_{\text{F}}^{2}}{\pi R^{2}}\frac{1}{\omega^{2}}\text{,}$$

where the Coulomb constant *k*_e_ = 1/4π*ε*_0_, and the effective permittivity of the background is *ε*_eff_. The imaginary part of the permittivity, Im(*ε*_SWNT_), can be obtained from the local plasmon property *Q* = {*ω*Re(*dε*_SWNT_/d*ω*)}/{2Im(*ε*_SWNT_)}. It is noteworthy that the surface plasmon dispersion relation of cylinders in the transcendental form can provide the exact solution of Re(*ε*_SWNT_) instead of Eq. (11) [18, 55, 56]. Experimentally, *λ*_p_ and *Q* can be measured by scattering near-field optical microscopy (SNOM) [19, 54, 57], while *R* can be obtained by AFM. The DC electron tunneling experiment can also measure *λ*_p_ if only Re(*ε*_SWNT_) is required [58], [59], [60], [61]. The method for determining the permittivity discussed here is applicable to other paramagnetic 1D metals because they support the Luttinger liquid [62].

For 1D semiconductors, however, the experimental determination of the permittivity of isolated 1D semiconductors is still lacking experimentally. The experimental techniques for 1D semiconductors should be explored. Note that optical identification of chirality indices of individual carbon nanotubes is possible via the reflection contrast spectroscopy [63, 64]. It is also noteworthy that a composite film of 1D materials becomes a 2D film of effective permittivity that can be characterized by reflection contrast-based ellipsometry and the conventional spectroscopic ellipsometry discussed in Section 3 [65, 66].

## 5 Conclusion and outlook

In this review, we have reviewed the basic concepts and recent progress in spectroscopic ellipsometry for 2D materials. Spectroscopic ellipsometry is one of the easiest ways to characterize material electronic structures via optical permittivity, and it can promote experimental efforts to discover new 2D materials. The well-established conventional spectroscopic ellipsometry techniques (Section 3.2) may be used to measure the optical permittivity of 2D materials experimentally, but the reflection contrast between a 2D material and substrate enables more straightforward and deterministic ellipsometry (Section 3.1 and 3.3), without any ambiguity originating from the fitting in the conventional ellipsometry.

We again emphasize that the technical challenges and issues discussed in Section 4 still need to be resolved. To summarize, (i) reflection contrast-based spectroscopic ellipsometry for thicker heterostructures should be developed. As shown in Section 3.3, applying the reflection contrast to ellipsometry enables the deterministic measurement of the permittivity for a 2D monolayer without fitting. The extension of this technique to thicker heterostructures will benefit the precise measurement of the material electronic structures. (ii) Spectroscopic ellipsometry, including broken inversion and time-reversal symmetry, should be explored. All ellipsometry discussed in this review considers only linear dielectric optical responses. Spectroscopic ellipsometry with external perturbations such as DC electric and magnetic fields can elucidate information of symmetry and electronic structures. (iii) Optical techniques to determine the permittivity of general 1D materials should be explored.

In addition to the advantages of ellipsometry in condensed matter physics studies, ellipsometry for the optical permittivity of low-dimensional materials also benefits their nanophotonic applications. Recently, optical communities have evinced great interest in integrating low-dimensional materials into nanophotonic devices because the exotic quantum properties of such materials can enable novel nanophotonic applications such as the nanophotonic routing of circularly polarized emission, 2D TMD lasers using photonic crystal cavities, graphene nanoantennas, molecular sensing, and novel Luttinger liquid-based infrared light sources. The tabulated data of the optical permittivity can be used as an input for the numerical simulations, including the finite-difference time-domain method (FDTD) and finite element method (FEM) to design nanophotonic devices. We believe that spectroscopic ellipsometry studies can promote the study of low-dimensional materials in condensed matter physics, material science, and nanophotonics.

## Supplementary Material

Supplementary Material
