# Supplementary material for: Spectroscopic ellipsometry for low-dimensional materials and heterostructures
Source: Nanophotonics. 2022 Apr 18;11(12):2811–25. doi: 10.1515/nanoph-2022-0039 (PMC11501394; doi:10.1515/nanoph-2022-0039)
Supplement: Supplementary file 1 — Supplementary Material [file j_nanoph-2022-0039_suppl.docx]

SeokJae Yoo* and Q-Han Park*

Supplementary Material: Spectroscopic ellipsometry for low-dimensional materials and heterostructures

**Reflection contrast in the 3D slab model**

Airy’s formula provides a reflection coefficient of a slab of refractive index *n*_2_ and thickness *d* (subscript 2) sandwiched by a superstrate (subscript 1) and a substrate (subscript 3) [1],

,

*r*_ij_ is called a Fresnel formula describing a reflection coefficient of a single interface between medium *i* and medium *j*. The phase across the slab is given by , where the *z*-axis is normal to the interfaces. Since the phase change across the slab is small, we can expand Eq. up to the first order,

.

We suppose the normal incidence of light, yielding with the wavenumber in free space $k_{0}=2\pi/\lambda$ and the wavelength of light *λ*. Also, refractive indices are given by *n*_1_=1 (air superstrate), *n*_2_=*n*_s_, and *n*_3_=*n*_sub_, respectively. Under the normal incidence, the Fresnel formula becomes a simple form,

$r_{pq}=\frac{n_{p}-n_{q}}{n_{p}+n_{q}}$.

Using Eq. , Eq. becomes

.

Here, we can find the first term in Eq. is the reflection coefficient of a bare substrate *r*_sub_. $\varepsilon_{s}=n_{s}^{2}$ is the permittivity of the slab. Then, we are ready to obtain reflectance of the slab $R_{s}=\left|r\right|^{2}$ with the assumption of a non-absorptive substrate, *i.e.* a purely real-valued *n*_sub_. Then, *R*_s_ is also written up to the first order of (*k*_0_*d*) as follows:

$\begin{matrix} R_{s}=\left(r_{sub}\right)^{2}-4r_{sub}\left(k_{0}d\right)\frac{\mathrm{Im}\left(\varepsilon_{s}\right)}{\left(1+n_{sub}\right)^{2}}+\left|2i\left(k_{0}d\right)\frac{\varepsilon_{s}-n_{sub}^{2}}{\left(1+n_{sub}\right)^{2}}\right|^{2} \\ \approx R_{sub}-4r_{sub}\left(k_{0}d\right)\frac{\mathrm{Im}\left(\varepsilon_{s}\right)}{\left(1+n_{sub}\right)^{2}} \end{matrix}$.

where the reflectance of a bare substrate is given by $R_{sub}=\left|r_{sub}\right|^{2}=\left(r_{sub}\right)^{2}$. Then, the reflection contrast becomes

$\delta_{R}\equiv\frac{R_{s}-R_{sub}}{R_{sub}}=-4k_{0}d\frac{1}{r_{sub}}\frac{\mathrm{Im}\left(\varepsilon_{s}\right)}{\left(1+n_{sub}\right)^{2}}=\frac{4k_{0}d}{n_{sub}^{2}-1}\mathrm{Im}\left(\varepsilon_{s}\right)$.

**Reflection and reflection contrast in the 2D sheet model**

Using a standard approach to obtain a Fresnel formula, one can easily calculate a reflection coefficient and reflection contrast in the 2D sheet model. Suppose an interface on the *x*-axis between medium 1 and 2 has a surface current density **J**=*σ***E**, where *σ* and **E** are the sheet conductivity and the electric field at the interface, respectively. *x* and *z* axes form a plane of incidence. For the p-polarization, the reflection coefficients are given by

$r=-\frac{k_{1z}k_{2}\eta_{1}-k_{1}k_{2z}\eta_{2}+k_{1z}k_{2z}\eta_{1}\eta_{2}\sigma}{k_{1z}k_{2}\eta_{1}+k_{1}k_{2z}\eta_{2}+k_{1z}k_{2z}\eta_{1}\eta_{2}\sigma}$,

where $\eta_{i}=\sqrt{\mu_{i}/\varepsilon_{i}}$ is the impedance of medium *i* whose permittivity and permeability are given by *ε*_i_ and *μ*_i_. The wavenumber *k*_i_ of medium *i* has a dispersion relation $\omega=ck_{i}=cn_{i}k_{0}$. *k*_iz_ is the normal (*z*) component of the wave vector. If media are non-magnetic, *i.e.* *μ*_i_= *μ*_0_, the expression, Eq. , becomes

$r=-\frac{\varepsilon_{2}k_{1z}-\varepsilon_{1}k_{2z}+k_{1z}k_{2z}\eta_{0}\sigma/k_{0}}{\varepsilon_{2}k_{1z}+\varepsilon_{1}k_{2z}+k_{1z}k_{2z}\eta_{0}\sigma/k_{0}}$.

Using the relation, $k_{iz}/k_{i}=\cos\theta_{i}$, with the angle of the plane wave propagation in medium *i* ($\theta_{i}$), we are also able to express solutions in the form

$r=-\frac{n_{2}\cos\theta_{1}-n_{1}\cos\theta_{2}+\eta_{0}\sigma\cos\theta_{1}\cos\theta_{2}}{n_{2}\cos\theta_{1}+n_{1}\cos\theta_{2}+\eta_{0}\sigma\cos\theta_{1}\cos\theta_{2}}$.

This is the result derived in Ref.  [2]. We can find that Eq. in the 2D sheet model is much simpler than Eq. in the 3D slab model. It is also possible to obtain the dispersion relation of 2D plasmons by an exchange $k_{jz}\to ik_{jz}$. By the exchange, a pole of Eq. occurs when the plasmon dispersion relation, $\varepsilon_{1}/k_{1z}+\varepsilon_{2}/k_{2z}=-i\eta_{0}\sigma/k_{0}$, is satisfied. Note that this relation is the same as Eq. (3) of Ref. [3]. This is widely used for graphene plasmons in infrared frequencies [3].

Under the normal incidence with *n*_1_=1 and *n*_2_=*n*_sub_, we can expand *r* up to the first order of $\eta_{0}\sigma$ as follows:

$r=\frac{1-n_{sub}}{1+n_{sub}}-\frac{2\eta_{0}\sigma}{\left(1+n_{sub}\right)^{2}}=r_{sub}-\frac{2\eta_{0}\sigma}{\left(1+n_{sub}\right)^{2}}$.

Eq. in the 2D sheet model corresponds to Eq. in the 3D slab model. The same approach can be used to derive the reflection contrast in the 2D sheet model.

**Reference**

[1] Yeh P., *Optical Waves in Layered Media* (Wiley-Interscience, 2005).

[2] Merano M., *Fresnel Coefficients of a Two-Dimensional Atomic Crystal*, Phys. Rev. A **93**, 1 (2016).

[3] Jablan M., Buljan H., Soljačić M., *Plasmonics in Graphene at Infrared Frequencies*, Phys. Rev. B **80**, 1 (2009).
